# Probabilistic seismic hazard assessment of southern part of Ghana

**DOI:** 10.1007/s10950-017-9721-x

**Published:** 2017-12-15

**Authors:** Sylvanus T. Ahulu, Sylvester Kojo Danuor, Daniel K. Asiedu

**Affiliations:** 1 Geophysics Division, Ghana Geological Survey, Accra, Ghana; 2 Department of Physics, Kwame Nkrumah University of Science and Technology, Kumasi, Ghana; 3 School of Earth Science, University of Ghana, Legon, Ghana

**Keywords:** Probabilistic seismic hazard assessment, Peak ground acceleration, Earthquakes, Ghana, Accra

## Abstract

This paper presents a seismic hazard map for the southern part of Ghana prepared using the probabilistic approach, and seismic hazard assessment results for six cities. The seismic hazard map was prepared for 10% probability of exceedance for peak ground acceleration in 50 years. The input parameters used for the computations of hazard were obtained using data from a catalogue that was compiled and homogenised to moment magnitude (Mw). The catalogue covered a period of over a century (1615–2009). The hazard assessment is based on the Poisson model for earthquake occurrence, and hence, dependent events were identified and removed from the catalogue. The following attenuation relations were adopted and used in this study—Allen (for south and eastern Australia), Silva et al. (for Central and eastern North America), Campbell and Bozorgnia (for worldwide active-shallow-crust regions) and Chiou and Youngs (for worldwide active-shallow-crust regions). Logic-tree formalism was used to account for possible uncertainties associated with the attenuation relationships. OpenQuake software package was used for the hazard calculation. The highest level of seismic hazard is found in the Accra and Tema seismic zones, with estimated peak ground acceleration close to 0.2 g. The level of the seismic hazard in the southern part of Ghana diminishes with distance away from the Accra/Tema region to a value of 0.05 g at a distance of about 140 km.

## Introduction

Ghana is situated on the southeastern margin of the West Africa Craton, far away from any active plate boundary. However, it has suffered damaging earthquakes as far back as 1615 when an earthquake was felt in Elimina along the coast of Cape Coast. Other major earthquakes that occurred in Ghana are shown in Table 1 (Junner 1941; Quaah 1980; Ambraseys and Adams 1986). Recent seismic activity is low, and the strongest earthquake since 1940 had a magnitude of 4.9 on 9 February 1969.

**Table 1:** Earthquakes with Mw ≥ 4.5 in Ghana during the past century (Junner 1941; Quaah 1980; Ambraseys and Adams 1986)

Town/district (country)	Date	Magnitude (Mw)	Agency
	(dd/mm/yyyy)		
North of Axim	18/12/1636	Mw 5.7	NNA
Offshore, west of Accra	10/07/1862	Mw 6.8	BGS
Accra District	12/03/1858	Mw 4.5	NNA
West of Accra District	13/08/1883	Mw 4.6	NNA
Offshore, west of Accra	22/06/1939	Mw 6.4	NNA
Ho District	20/11/1906	Mw 5.0	NNA
Near Akosombo District	11/03/1964	Mw 4.7	AKO
Offshore, east of Accra	09/02/1969	Mw 4.9	USG
Accra District	06/03/1997	Mw 4.7	ISC

**Fig. 1:**
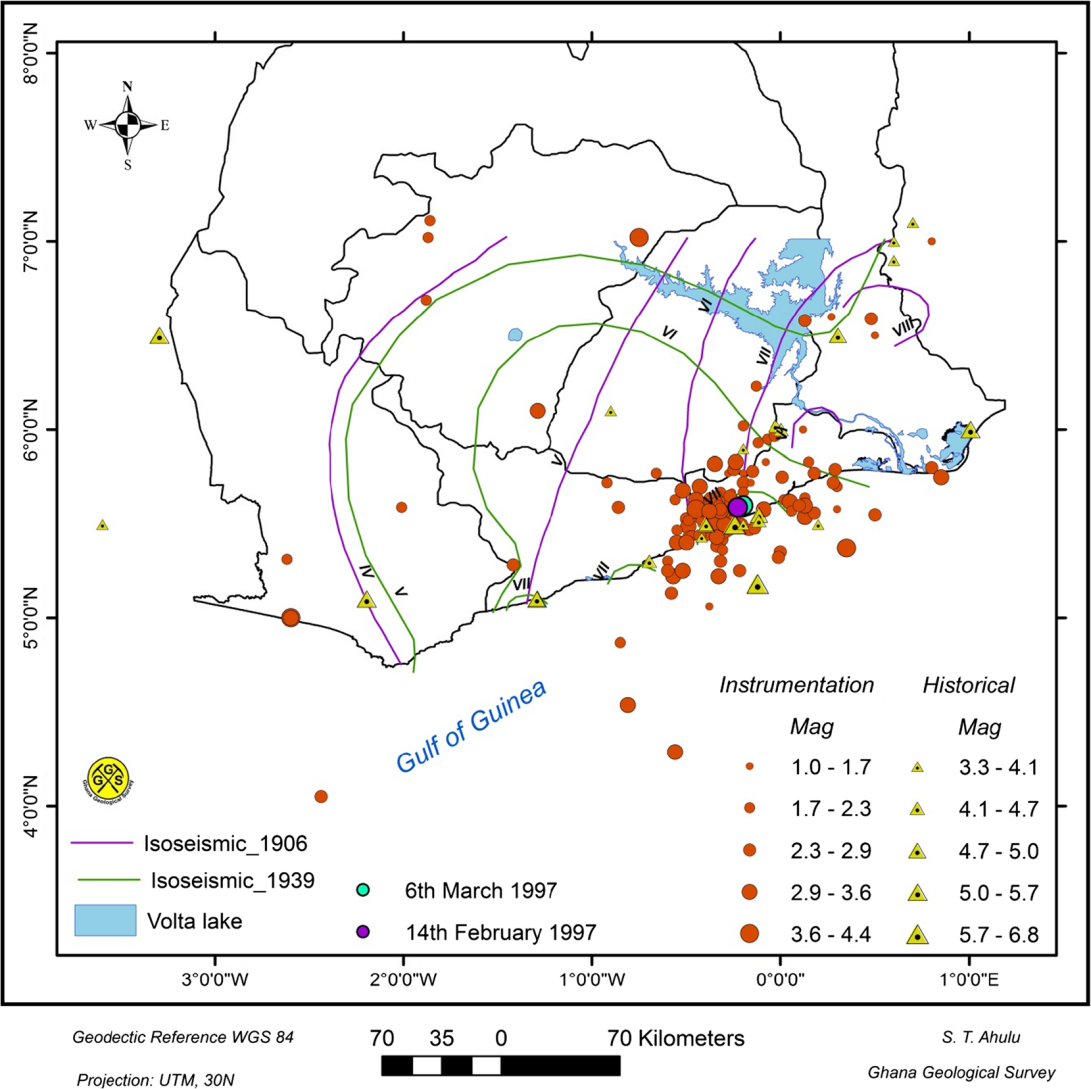
Seismicity of southern Ghana. Also shown are the isoseismal lines prepared for the 20 November 1906 and 22 June 1939 events. The intensity data were based on the Modified Mercalli Intensity scale (Junner 1941)

West Africa is adjacent to an inactive section of a transform fault related to the opening of the Atlantic Ocean. In view of the concentration of recent seismic activity in southeast Ghana (Fig. 1), the underlying forces are not sought in the globally active forces but rather in the local domain. The forces which cause seismic events in southeast Ghana are attributed to two major active faults: the Coastal Boundary Fault and the Akwapim fault zone. The Akwapim fault zone comprises a northeast–southwest running system of faults.

Earthquakes in Ghana are concentrated in the southern part of the country where the network of seismic recording stations is installed. However, smaller earthquakes outside of the network often go unnoticed.

The study seeks to introduce the probabilistic approach in seismic hazard assessment of the southern part of Ghana where seismic activity is quite high. This area includes the Accra region, which is experiencing rapid urbanisation but not considering seismic hazard in land use planning. The uncontrolled use of land for building has made it possible to develop sites that are vulnerable to earthquake hazard. Seismic hazard had previously been assessed by Amponsah et al. (2009) where they used the deterministic method for the capital city of the country, Accra Metropolitan Area. Kumapley (1996) revised the four seismic zones proposed by Anon. (1988), which were based on the 1939 earthquake isoseismal lines and assigned horizontal ground acceleration values. For these reasons, a revised assessment of hazard is urgently needed.

Recent development in seismic hazard assessment and its application to earthquake engineering indicated clearly that both the probabilistic and deterministic methods are equally important (Bommer 2003). The deterministic approach is essentially based on a worst case scenario where the event occurs close to the site. However, the approach does not provide any indication of how likely it is for this event to occur. Probabilistic Seismic Hazard Assessment (PSHA) takes into account the expected ground motions likelihood since it provides the probability of exceeding a specific ground motion level whereas the ground motions applied by Deterministic Seismic Hazard Assessment (DSHA) are associated with an unknown probability of exceedance. Seismic hazard assessment estimated using the probabilistic method handles various uncertainties whereas the deterministic approach generates distinct values of a ground motion parameter for a specific scenario.

## Seismicity in Ghana

Major earthquakes occurred in Ghana in the following years: 18 December 1636, where workers in a gold mine in Axim in the western region of the country were buried and several buildings collapsed; 10 July 1862, where the capital city of Accra was most affected, with cracks in important buildings; and 22 June 1939, which was the most destructive earthquake (Junner 1941; Quaah 1980; Ambraseys and Adams 1986).

Prominent among them is the 22 June 1939 earthquake with a magnitude of 6.5. The event shook the entire country (isoseismal lines in Fig. 1) and caused major destruction and loss of lives. The greatest damage to life and property occurred in Accra, where 16 people were killed and 133 injured. Hundreds of thousands of pounds of damage was caused to buildings. Focal mechanism studies of this earthquake led Yarwood and Doser (1990) and Kutu et al. (2013) to report that strike-slip faulting that occurred parallel to the coast of Ghana may have been responsible. The 11 March 1964 earthquake of magnitude 4.7 on the Richter scale occurred not far from the multi-million dollar hydro-electric dam at Akosombo. The 14 February 1997 earthquake, measuring 4.7 on the Richter scale, was felt in Accra. The 1997 event also had an aftershock on 6 March 1997 of magnitude 4.1 (Fig. 1). Parameters of the 1906, 1939 and 1969 events were reassessed by Amponsah et al. (2012) who obtained magnitude values of 5.0, 6.3 and 4.8 respectively, using a formula adopted and modified from Kanai (1983).

### Earthquake catalogue

**Fig. 2:**
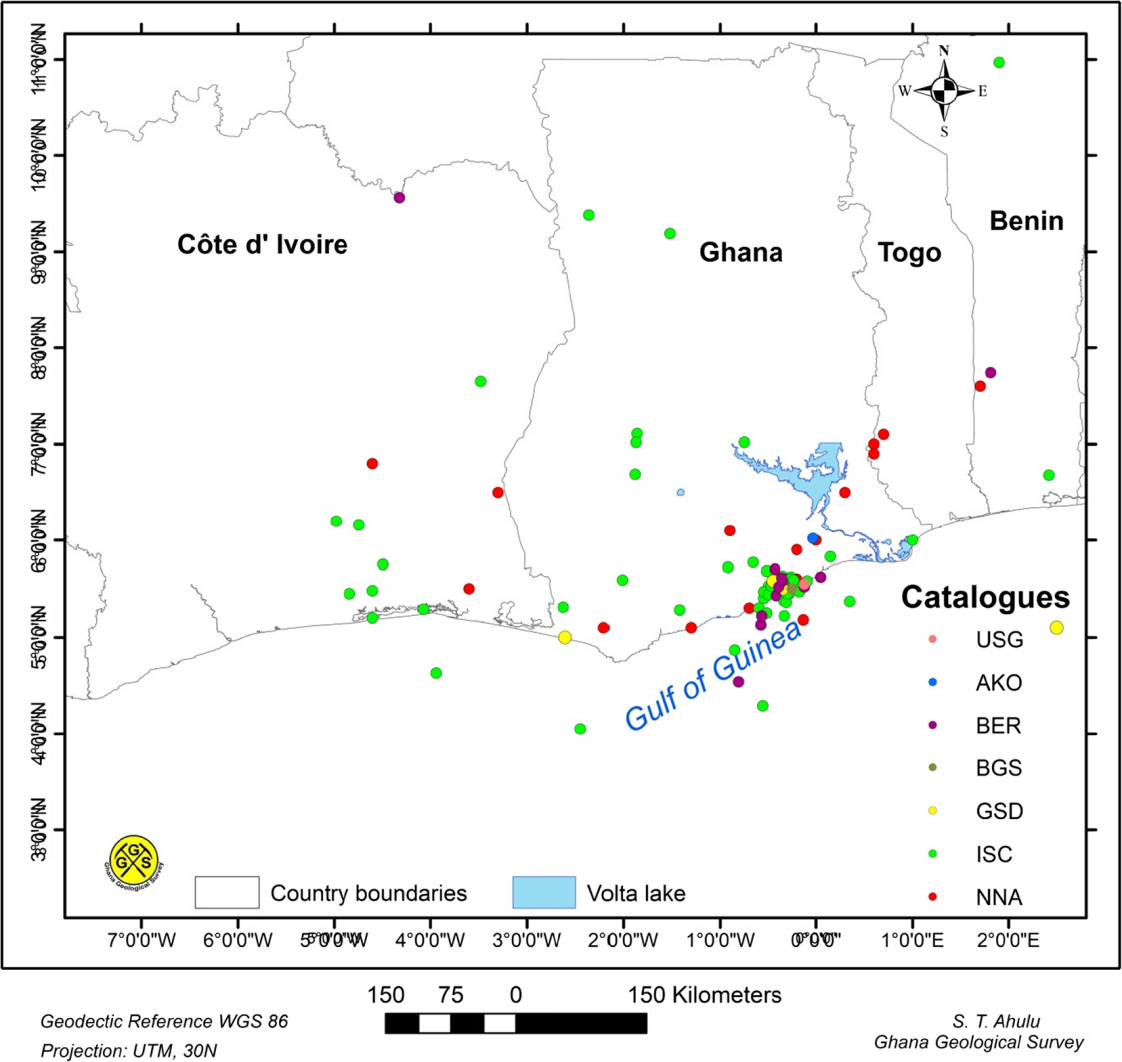
Seismicity in the catalogue compiled by Musson (2014) and used in this study. Data sources: USG—United States Geological Survey, AKO—Akoto and Anum (1992), BER—Bertil (1991), BGS—British Geological Survey, GSD—Geological Survey Department, ISC—International Seismological Centre, NNA—Ambraseys and Adams (1986)

The earthquake data used in this study (Fig. 2) were obtained from the earthquake catalogue compiled by Musson (2014). The data were based on earthquakes between latitudes 4° N–8° N and longitudes − 4° W–2° E. In his compilation, Musson (2014) obtain data from different sources and cover the period 1615–2009. He went on to homogenise the earthquake magnitude values to the moment magnitude (Mw), which is a direct indicator of the co-seismic deformation (Boore and Joyner 1984; Joyner 1984).

The Poisson model of earthquake occurrence, which assumes that events in the catalogue are independent (Bender and Perkins 1987), was adopted. To ensure compliance with the model, the catalogue was de-clustered using the de-clustering algorithm of Gardner and Knopoff (1974), which is implemented in the AFTERAN programme (Musson 1999), and distributed in the OpenQuake software (Marco et al. 2016). In this case, an initial catalogue of 127 events was left with a sub-catalogue of 64 events after de-clustering.

**Fig. 3:**
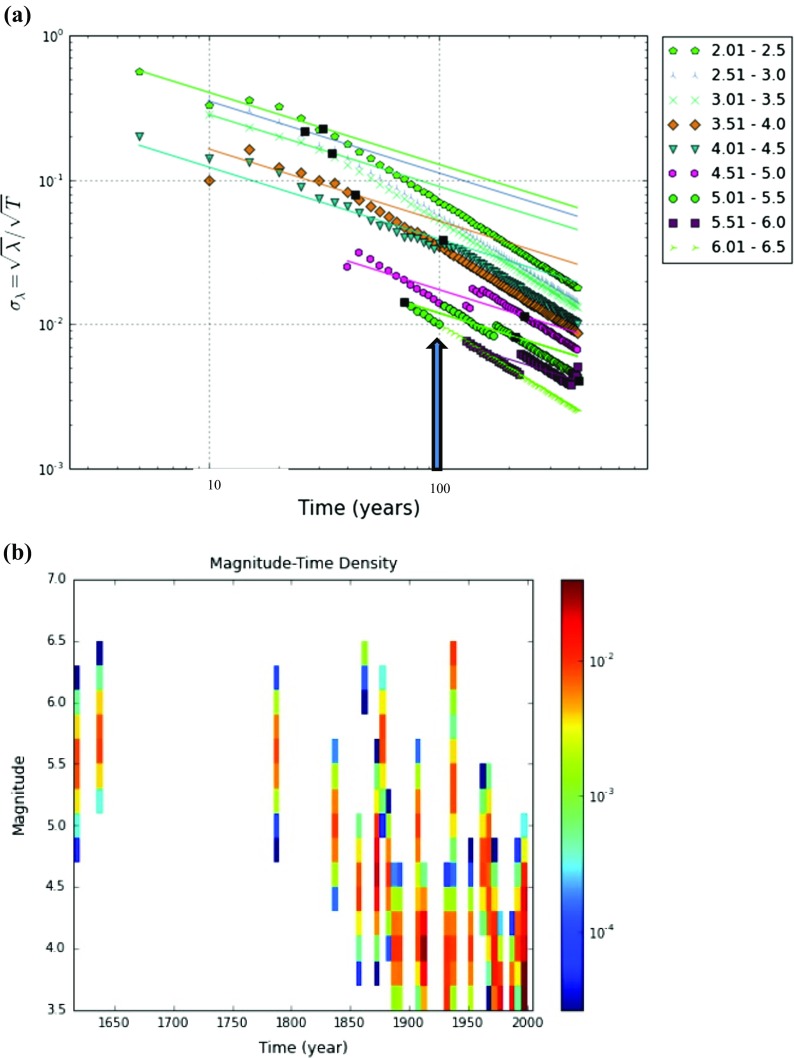
Results of completeness estimation conducted using **a** Stepp (1971) and **b** Magnitude–time–density methodology. The black arrow indicates the observational period, ∆*T*

Usually in seismic hazard assessment, a catalogue that is used for the calculation of recurrence parameters needs to be assessed in terms of its completeness. Magnitude of completeness is defined as the lowest magnitude above which 100% of the events in space–time volume are detected (Mignan and Woessner 2012; Woessner and Wiemer 2005; Rydelek and Sacks 1989). A statistical method in an algorithm by Stepp (1971) and implemented in OpenQuake was used to determine the time variation in completeness. This method is based on the assumption that the earthquake distribution is Poissonian. This process is fully described by the mean occurrence rate *λ*—the variance of which is given by *σ* ^2^ = *λ*/∆*T* (∆*T* is the observational period). The completeness is estimated by visual identification on the graph in Fig. 3a, by the departure of the data *σ* from the expected slope of the plotted events with increasing observational periods as indicated by the black arrow. Similarly, Fig. 3b shows the estimated completeness by visually identifying from the graph the maximum observational period with the corresponding magnitude. The analysis indicates that the harmonised catalogue is complete for the same magnitude Mw ≥ 4 for the period between 1850 and 2009. The accuracy of the completeness magnitude depends on the magnitude and time interval considered, and a degree of judgement is often needed to determine the time at which the rate deviates from the expected values. Thus, any remaining events with Mw < 4 were also excluded from the working catalogue; hence, the working catalogue was left with 33 events.

### Tectonic provinces

**Fig. 4:**
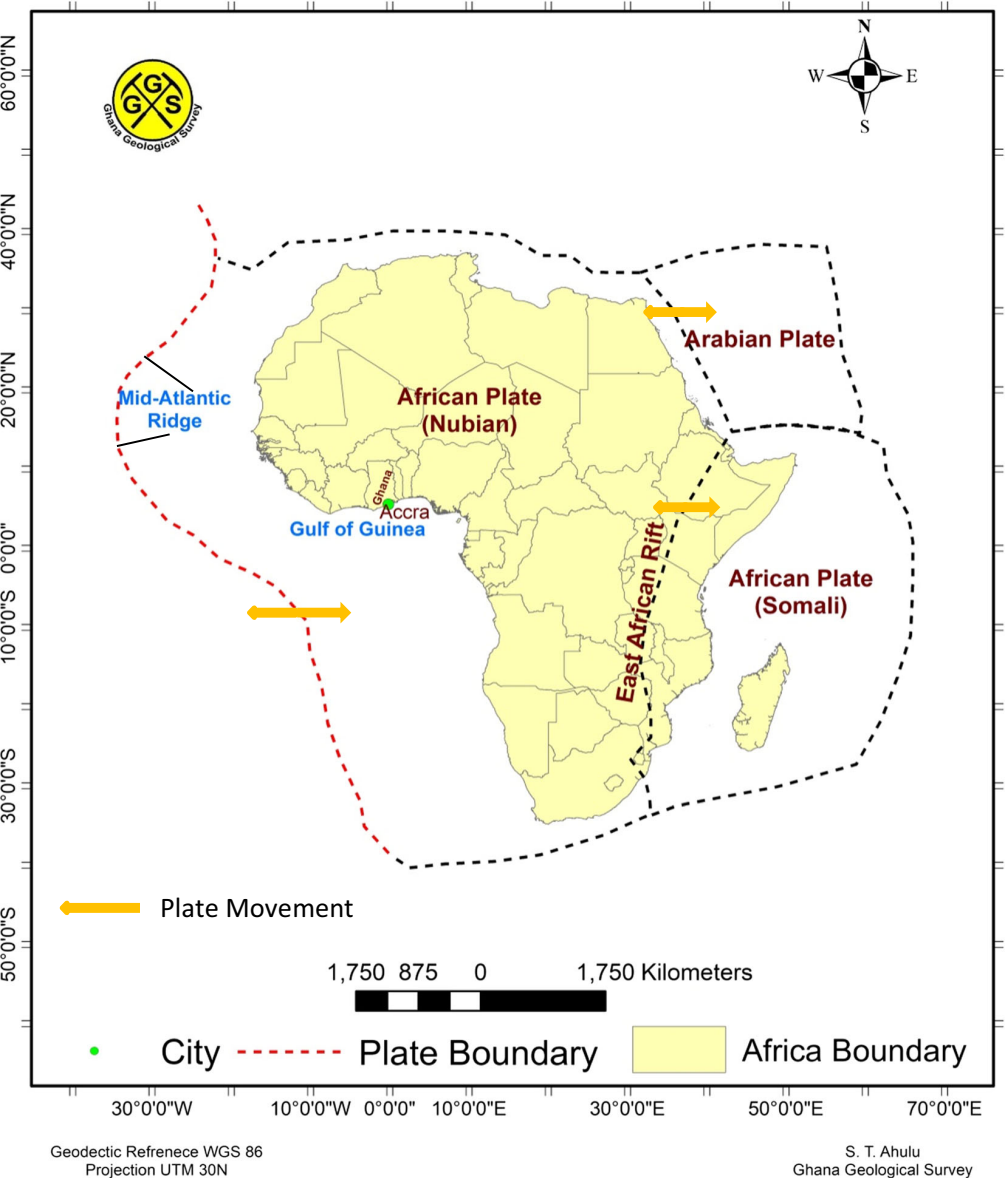
Plate tectonic regions around Africa

Figure 4 shows the position of Ghana within the Africa plate. Ghana is in the West Africa zone within the western part of the plate. The divergent movement between the American and Africa plates forms the Mid-Atlantic ridge. It is a constructive plate boundary located along the floor of the Atlantic Ocean, and is part of the longest mountain range in the world. The ridge has an average spreading rate of about 2.5 cm per year (USGS 2011).

The rate of movement on the western side of the plate is relatively very slow, 2.0–15 mm/year (Hartnady and Benouar 2007). This slow movement makes the reoccurrence period of large earthquake large. In this case, most of the microseismic activities around Ghana are from intra-plate movement.

Africa is experiencing an intra-continental rifting along the East African Rift system. The rift is a narrow zone that is developing a divergent tectonic plate boundary, in which the African Plate is in the process of splitting into two tectonic plates, referred to as the Somali Plate and the Nubian Plate (Fernandes et al. 2004).

This East African Rift system controls most tectonic features both in Eastern and Southern Africa. This geological structure extends over a distance of approximately 4000 km from the triple junction in the Afar region to the less matured continental rifting in the south (Midzi et al. 1999).

### Seismotectonics of Ghana

#### Akwapim fault

**Fig. 5:**
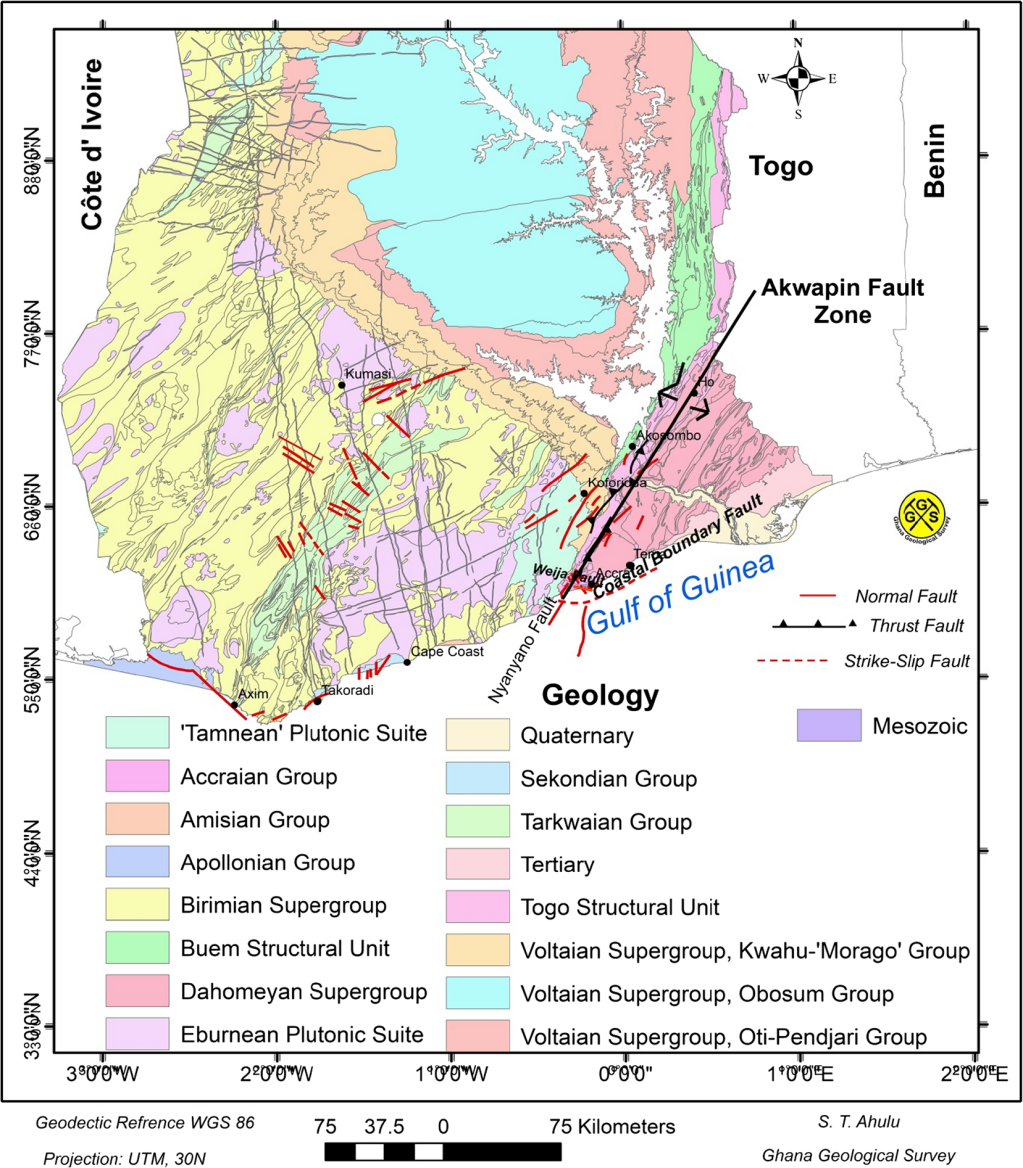
Tectonic setting of the southern portion of Ghana

Most of the recent earthquakes or tremors in Ghana are located along two major active fault zones, which are the Coastal Boundary Fault zone and the Akwapim fault zone (Fig. 5).

The Akwapim fault, which trends in a northeasterly direction from just west of Accra, is part of the Akwapim fault zone in which more recent faulting has occurred along an ancient line of thrust boundaries between the Birimian, at the west of the Togo Series, and the Dahomeyan on the eastern part of the Togo Series. The Dahomeyan is composed of a variety of medium-grade metamorphic rock units (GSD and BGR 2009). This Akwapim fault zone stretches northeastwards through Kpong, Ho, and into the Republic of Togo and Benin (Fig. 5). Recent large-scale mapping in the southern part of the Akwapim fault zone (Muff and Efa 2006) shows that at a later stage, the Akwapim–Togo Belt was subjected to a block-tectonic style of deformation, and that many normal faults of local extent have developed in recent times (Fig. 5).

#### Coastal Boundary Fault

The West African continental margin was formed during the opening of the Atlantic Ocean. Basic magmatism was associated with the initial period of opening. Flanking sedimentary troughs developed near the continental margin with subsidence continuing from the Jurassic to the present. The sediment troughs are defined by faults separating individual crustal blocks, which continue to be active throughout the history of subsidence, the most active of these being the Coastal Boundary Fault (Blundell and Banson 1975). It strikes approximately north 60°–70° east at a distance of 3–5 km from the coast and down throws the block south of it for several kilometres.

The Coastal Boundary Fault is the northern boundary of a basin filled with sediments of Upper Jurassic to recent age. Blundell (1976) also wrote that the Coastal Boundary Fault forms the northern margin of the Keta Basin. He further explained that the fault could probably be active throughout the entire time of deposition. To the west of Accra, the fault bends to strike E°W and intersects with the Nyanyanu fault in the Akwapim fault zone.

A recent review of geological and instrumental recordings (Van Landewicjk 1980; Akoto and Anum 1992; Essel 1997; Amponsah 2004) shows that earthquakes have occurred in the past and are still likely to occur within the vicinity of the intersection of the Akwapim fault zone and the Coastal Boundary Fault. There are a number of other faults in the acute angle between these two major faults, the most important of which is the Weija fault striking WNW (Fig. 5). Numerous active faults have been mapped during the course of foundation investigation studies in and around Accra. The area within the acute intersection has high seismicity (Fig. 1). Bacon and Quaah (1981) reported that the concentration of epicentres in the western part of Accra at the junction of the two major fault systems indicates that the faults around Weija are likely active.

Amponsah et al. (2012) support the findings of these faults to have been likely active by attributing their activeness to tectonic settings related to tectonic dynamics. According to them, the presence of high-to-moderate-angle neotectonic normal faults in the Akwapim Range may indicate that tectonic movement is still ongoing, and therefore could make faults from SW of the Akwapim Range around Weija likely active. Freeth (1978) considered plate tectonic forces to be responsible for tectonic activity in West Africa. He suggested that the northward movement of the African plate over the ellipsoid-shaped surface of the Earth causes extensional stresses, which is ongoing still now. According to him, these extensional stresses may be contributing to seismic activity within the West Africa zone and therefore could make some faults within the zone likely active. Previous studies (Tevendale 1957; Burke 1969b; Bondesen and Smit 1972; Ahmed et al. 1977) also related seismic activities with tectonic elements in SE Ghana.

## Seismic source characterisation

### Area source zones

Major inputs into a probabilistic seismic hazard analysis include seismic source zones, seismic source parameters and ground motion prediction equations. Each of these inputs is discussed in the following sections.

The area source zones are delineated by defining the potential seismic source zones, zones which describe the potential locations of future earthquakes. These zones are usually associated with active geological or tectonic features (e.g. faults) and seismicity.

However, it is very difficult to identify individual fault zones that are seismically active. This is due to the fact that the region is not properly monitored due to lack of stations, and inability to properly identify active faults through better-detailed Quaternary mappings at a suitable scale. In this case, the characteristic elements, such as fault geometry, slip rate of faulting during the recent geologic period and fault segmentation length, are not available. Mavonga and Durrheim (2009) reported areas where there are no available documents on location of potential faults that may cause a scenario earthquake; quantification of the seismic hazard is based mainly on the seismic and geological history available in the area.

Nevertheless, our current knowledge of the tectonics and seismicity of the study area as shown in Figs. 2 and 5 is good enough to delineate area source models within which earthquake characteristics may be assumed to be uniform.

**Fig. 6:**
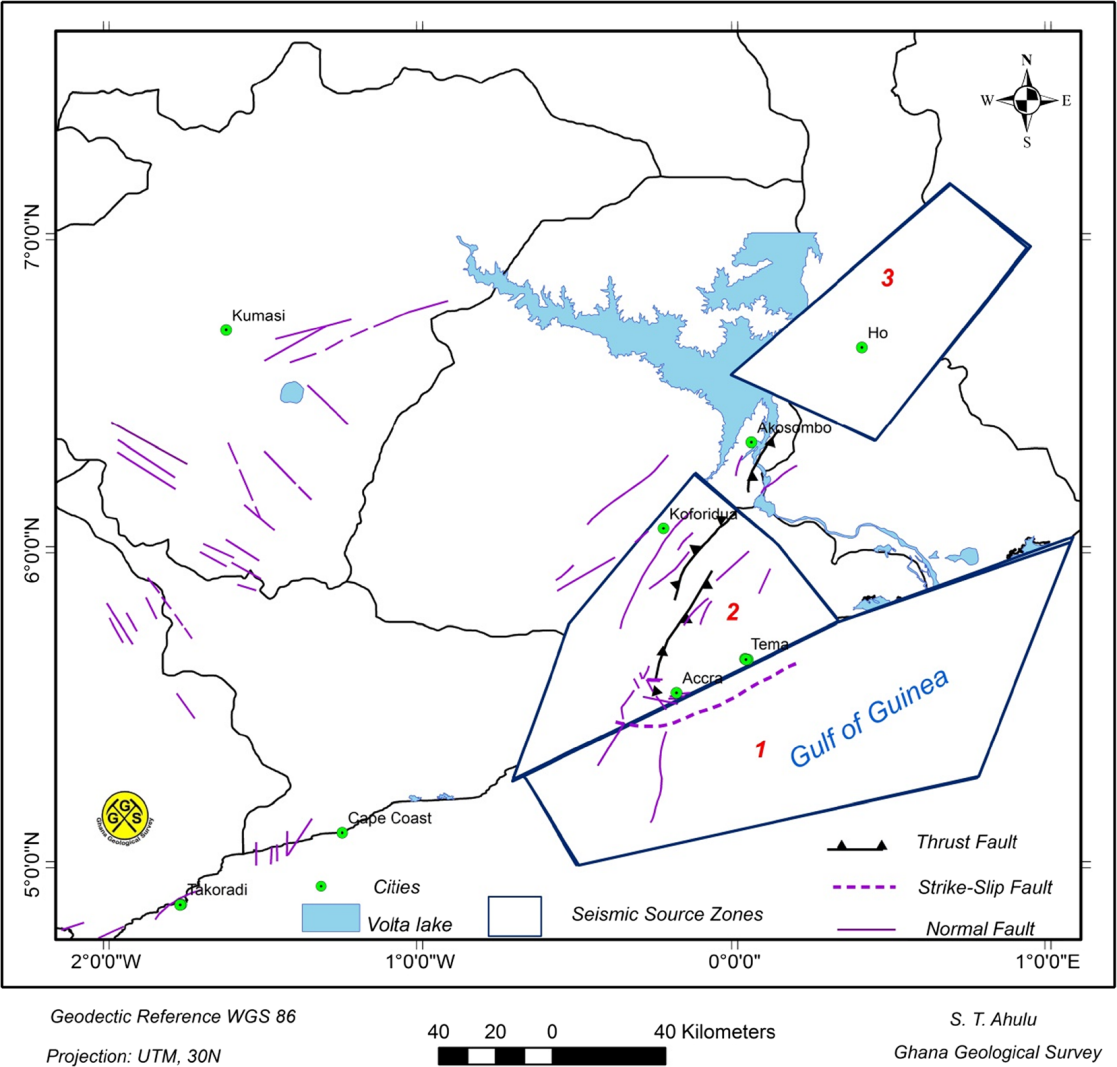
Seismic source zones marked as polygons (1 = off shore—Jurassic. 2 = Accra region—Neoproterozoic. 3 = NNE of Ho region—Neoproterozoic)

Three seismic source zones were identified as the main contributors to damaging earthquakes in the southern part of Ghana (Fig. 6).

1. Offshore zone in the Gulf of Guinea—Jurassic.
2. Accra region—Neoproterozoic (Pan-African Mobile Belt)
3. NNE of Ho region—Neoproterozoic (Pan-African Mobile Belt)

### Seismic source parameters

Seismicity parameters are determined for each seismic source zone (Table 2). The seismic characteristics within the study area were modelled as a Poisson process, based on engineering seismology standard assumptions. These assumptions are based on Poisson distribution conditions where events are independent and mean occurrence rate *λ* relates to seismic activity rate or earthquake occurrence rate.

**Table 2:** Seismicity parameters for the area source zones, where M_min_—lower-bound magnitude; M_max_—maximum expected upper-bound magnitude; Beta (*β*)—*b* value × ln (10); lambda (*λ*)—annual number of earthquakes above the lower magnitude bound

Source zones	M_min_	M_max_	Beta (*β*)	Lambda (*λ*)
1	4	7.3	1.70	0.104
2	4	5.2	1.70	0.032
3	4	5.5	1.70	0.024

The parameters used to determine the characteristics of each seismic source zone are as follows:

1. Average rate of occurrence or mean seismic activity rate *λ*
2. Level of completeness of the earthquake catalogue (M_min_)
3. Maximum possible earthquake magnitude (M_max_)
4. Gutenberg–Richter (1954) “*b* value” (which indicates the relative number of large and small earthquakes, *β* = *b* (ln10)
5. Focal depth

The completeness parameter of Mw = 4 was calculated using the OpenQuake software, and the process followed is described in Sect. 2.1. Furthermore, Mw = 4 was selected as the lower magnitude bound (M_min_) because earthquakes with Mw < 4 are considered unlikely to cause damage, even to houses that are poorly designed and built.

The seismic code software developed by Kijko and Smit (2012) was used to calculate the *b* value and Lambda (*λ*), using the catalogue of 33 events. Hence, 17 events were for zone 1, 11 events for zone 2 and 5 events for zone 3 respectively. Maximum standard deviation obtained in the calculation of the *b* value was ± 0.09. The *b* value is expected to be regionally stable with variations less than the uncertainty limits, while the activity rate *λ* is liable to vary substantially from one seismic source zone to another. The *b* value obtained in this study was determined using the combined events from all the zones (33 events) as it was assumed that all the zones are governed by almost the same tectonic setting. Hence, the *b* value is regionally stable. However, lambda (*λ*) varies significantly and was thus calculated separately for each zone by considering the number of events identified for each zone in proportion to the lambda (*λ*) value calculated, using the catalogue of 33 events.

The maximum credible magnitude M_max_ was obtained using M_max_ = M_max_^obs^ + 0.5 (Gupta 2002), where M_max_^obs^ is the largest observed magnitude. Although the catalogue used for this study covers over century duration, and it is complete for events of magnitude Mw ≥ 4, the value of an important parameter (Max^obs^) which was estimated on the basis of the observed maximum magnitude from the catalogue could be underestimated. This is because the African plate boundaries are generally characterised by slow relative movement (~ 2 to ~ 15 mm/year), and therefore large earthquakes such as (Max^obs^) have extremely long recurrence periods (Hartnady and Benouar 2007).

The accuracy of the focal depths is generally poor due to the sparse station coverage and spacing. However, microseismic studies indicate that the earthquake foci are generally between depths of 1 to 16 km for SE part of Ghana (Bacon and Quaah 1981). Consequently, we used a value of 10 and 15 km in the logic tree formalism.

## Ground motion models

The selection of appropriate attenuation models depends mostly on the ground motions of a geographical region. However, this selection is difficult for regions where no or little observed strong-motion data is available such as in Ghana. Therefore, it is often preferable to use well-constrained models, developed using data from other regions of similar tectonics, than to predict motions using local models that are poorly constrained (Douglas 2007). Given this lack of strong motion data and attenuation equations for Ghana, it was decided to use appropriate models from other regions in this study.

Ghana is within the Sub-Saharan Africa region, and is in a stable intra-plate region, characterised by a relatively low level of seismic activity, with randomly distributed earthquakes in space and time. The only parts of Sub-Saharan Africa that do not display the characteristics of an intra-plate region are the East African Rift System and the Cameroon volcanic line, where earthquakes are associated with active fault zones and volcanic activity (Kubanza et al. 2006).

It is recommended that hazard be represented by several attenuation equations with the median or mean curve rather than a single curve. In this way, both the uncertainty and the central value of the hazard are represented and may be considered for mitigation decision (Risk Engineering 2007). However, in this present study, we used some of the well-accepted ground motion prediction equations (GMPEs) which were developed for other regions of the world but having similar seismic attenuation characteristics of the study area. Hence, using these GMPEs reduces the uncertainty associated to lack of data to generate attenuation equations of the study area.

Four attenuation relations were considered for the estimation of the ground motion that is likely to be experienced within the study area: Allen (2012), Silva et al. (2008), Campbell and Bozorgnia (2008) and Chiou and Youngs (2008). The equations by Allen (2012) and Silva et al. (2008) were derived using data from South Eastern Australia and Central and eastern North America respectively. These equations are for a stable continental area and thus assumed to be most suitable for Ghana. The North American attenuation equations (Silva et al. 2008) are the best known and most frequently used attenuation formulas for stable continental regions (Kijko et al. 2002). The other equations selected were prepared by Campbell and Bozorgnia (2008) and Chiou and Youngs (2008), and were derived for active-shallow-crust regions. These equations were selected because this stable region is likely to have faulting observed in the active region. This is due to the fact that the prevalent active normal faulting and thrust faulting within the Akwapim fault zone in the study area are similarly experienced in the active region. Hence, the consistency of both regions likely experiencing similar active faulting systems makes the attenuation model for this stable region comparable to that of the active region. Susana and Fonseca (2007) applied a similar concept in their studies on the assumption that both regions (active continental region and stable continental region) are experiencing a similar active faulting system and therefore their crust transmits seismic energy efficiently.

**Fig. 7:**
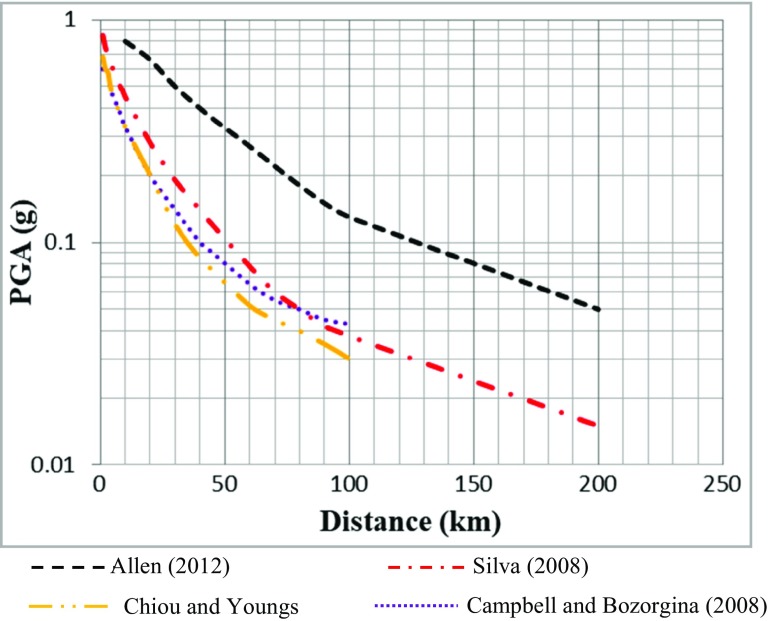
Attenuation curves obtained for South Eastern Australia (Allen 2012), Central and eastern North America [Silva et al. 2008] and worldwide active-shallow-crusts (Campbell and Bozorgnia 2008) and (Chiou and Youngs 2008)

Attenuation curves for the four equations are shown in Fig. 7 and show very different predicted acceleration values at all rupture distance. The range of magnitude values of earthquakes used to derive the four equations started with a minimum magnitude greater than or equal to Mw = 4.

## Logic-tree

Uncertainties in the models used for seismic hazard assessment make the selection of a seismic hazard model difficult. These uncertainties includes aleatory variability that is associated with GMPEs and is generally represented by the standard deviation of the logarithmic residuals of the predicted parameter, and the epistemic uncertainties that reflects the incomplete knowledge of seismicity, rupture characteristics and seismic energy excitation. Epistemic uncertainties also include the characteristics of the seismic source zones (be these area zones or specific faults), the model for the recurrence relationship, the maximum earthquake magnitude and GMPEs.

**Fig. 8:**
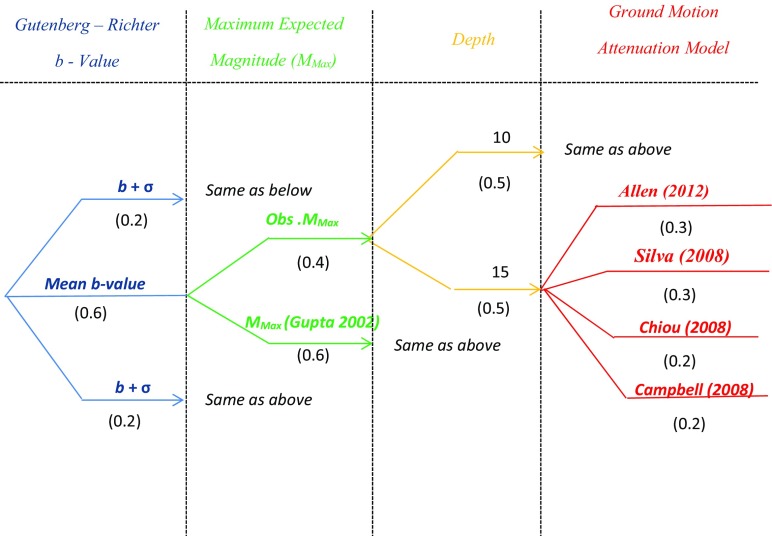
Logic-tree used for hazard calculation. The weight of each branch is shown in parentheses

The use of the logic-tree approach allows characterisation of epistemic uncertainties in various models by including alternative models in the analysis (Budnitz et al. 1997; Stepp et al. 2001; Bommer et al. 2005). A logic-tree consists of a series of nodes and branches, and these branches denote different models (Fig. 8). A subjective weight is assigned to each of these branches depending on the likelihood of being correct.

The sum of the weights for all the branches at a particular node should be equal to unity. The weight of each terminal branch of the logic-tree can be obtained by multiplying the weight of all the branches leading to it.

The present study considers one type of source model and four different ground motion prediction equations (Table 3).

**Table 3:** Characteristics of the considered GMPEs

GMPE	Region	Magnitude type and range (Mw)	Distance definition (km) γ_rup/JyB_	Spectral period PGA	Site conditions	Faulting mechanism	Horizontal component definition
Allen (2012)	South Eastern Australia	(4.0–7.5)	(> 400)	(0.1–10)	Hard-rock sites V_s_30 820 m/s	Strike-slip/ Normal slip/ Reverse slip	Average horizontal component
Silva et al. (2008)	Central and eastern North America	(4.5–7.5)	(1–400)	(0.1–10)	Hard-rock sites Vs30 2830 m/s Vs30 2310 m/s	Strike-slip/ Normal slip/ Reverse slip	Average horizontal component
Campbell and Bozorgnia (2008)	Worldwide	(4.0–8.5)	(1–200)	(0.1–10 s)	Hard-rock sites V_s_30 1100 m/s	Strike-slip/ Normal slip/ Reverse slip	Average horizontal component
Chiou and Youngs (2008)	Worldwide	(4.0–8.5)	(1–200)	(0.1–10 s)	Hard-rock sites V_s_30 1130 m/s	Strike-slip/ Normal slip/ Reverse slip	Average horizontal component

Two different maximum magnitudes were considered for each area source; the maximum historical magnitude M_max_^obs^ and the previous increased by 0.5 units M_max_ + 0.5 (Gupta 2002).

## Estimation of hazard and results obtained

### Brief theoretical background

The seismic hazard calculation at a given site due to multiple earthquake source zones can be represented by the following equation (Reiter 1990) which uses the total probability theorem to calculate the probability of a ground motion (e.g. peak ground acceleration, velocity or displacement) being exceeded at a given site.

$$ H(a)=\sum \limits_{i=1}^n{\nu}_i\iint P\left[A>a|m,r\right]{f}_{M_i}(m){f}_{R_i}\mid {M}_i\left(r,m\right) dmdr $$

where

H (a): is the annual frequency of earthquake that produces ground motion with amplitude *A* greater than *a.*
*v*_*i*_: is the annual rate of occurrence of earthquakes (with magnitude greater than M_min_) in the *i*th source area.
P [*A*>*a* | m, r]: is the probability that an earthquake of magnitude *m* at distance *r* produces a ground motion amplitude *A* at the site that is greater than *a.* It is obtained from the cumulative lognormal distribution with specified standard deviation σ as

$$ P\left[A>a|m,r\right]=1-\frac{1}{\sqrt{2\Pi}}\frac{1}{\sigma}\underset{-\propto }{\overset{\ln a}{\int }}\exp \left[-{\left(u-y\right)}^2\frac{1}{2{\sigma}^2}\right] du $$

where

*y* = C_1_ + C_2_ M + C_3_ ln (R + C_5_) + C_4_ R + ɛ ɛ ≈ N (0, σ) for each source.

C_1_, C_2_, C_3_, C_4_, and C_5_: are empirical constants.
M: is the magnitude of earthquakes.
Ɛ: is a random error which has a normal distribution with mean 0 and variance σ^2^.
$$ {f}_{M_i}(m) $$
is the probability density function of earthquake magnitude. It depends upon the earthquake magnitude recurrence model. Often ${f}_{M_i}$ (m) is assumed to be an exponential truncated at lower and upper limits by M_min_ and M_max_.
$$ {f}_{R_i}\mid {M}_i\left(r,m\right) $$
is the probability density function of earthquake-site distance. It depends upon the geometry of earthquake source, which usually takes the form of a point, line or bounded surface.

The occurrence of earthquakes is assumed to follow the Poisson probability density function. Therefore, the probability of exceedance *r* (*a*) of the ground motion *a* is often expressed by the following equation:

$$ r(a)=1-\exp \left(- TH(a)\right) $$

where

*T*: is the time period (number of years) for which we want to know the probability
*H* (*a*): is the annual rate of exceedance of ground motion *a*
1*/H* (*a*): is the return period

The hazard calculations for rock site condition were done using the OpenQuake 2016 software (Marco et al. 2016) for 10% chance of exceedance in 50 years. The OpenQuake 2016 software incorporates the epistemic uncertainty into the hazard calculations and also integrates across the aleatory variability within the hazard calculations.

**Fig. 9:**
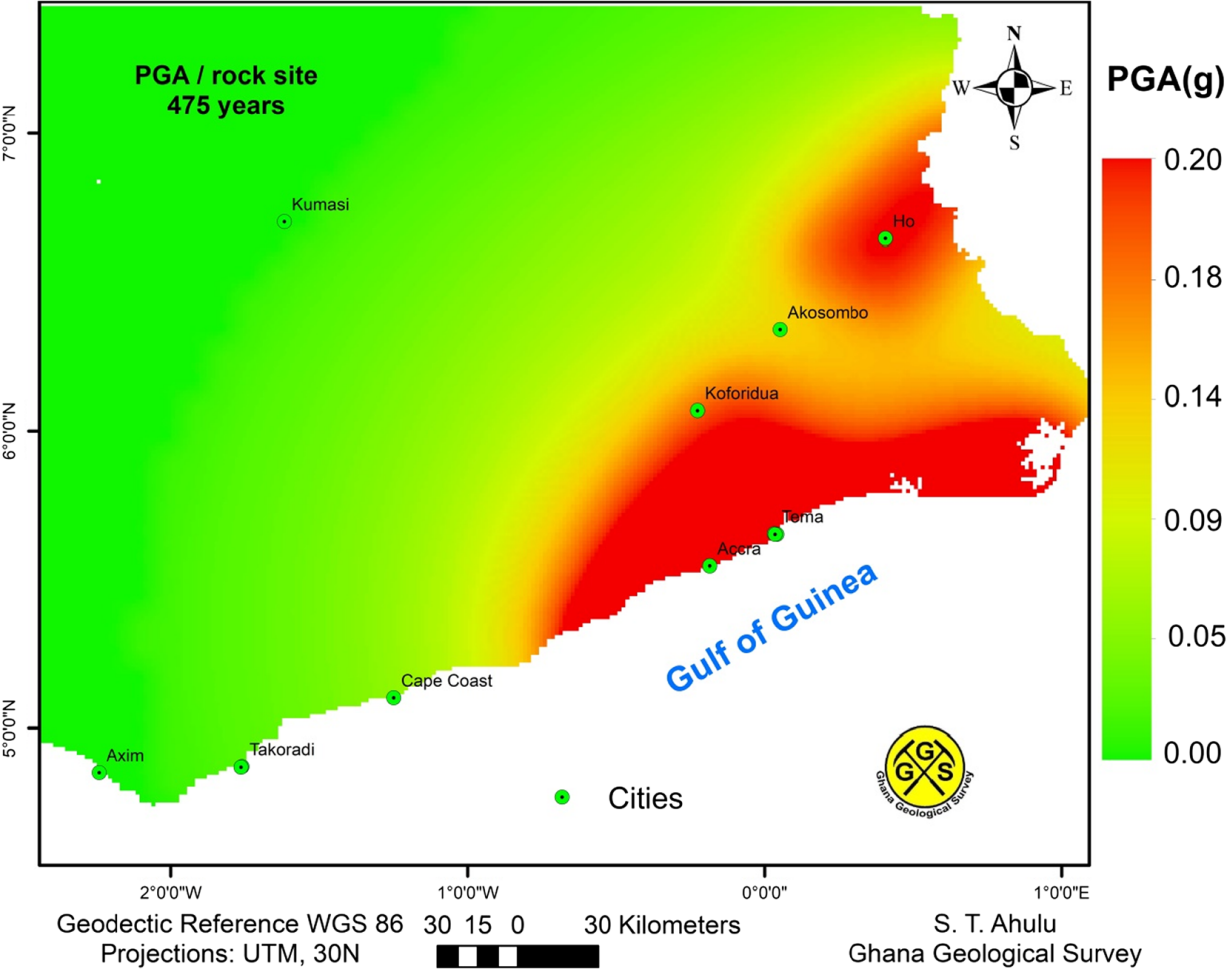
Distribution of mean PGA values (in unit g) in the southern part of Ghana computed for 10% chance of exceedance in 50 years

**Fig. 10:**
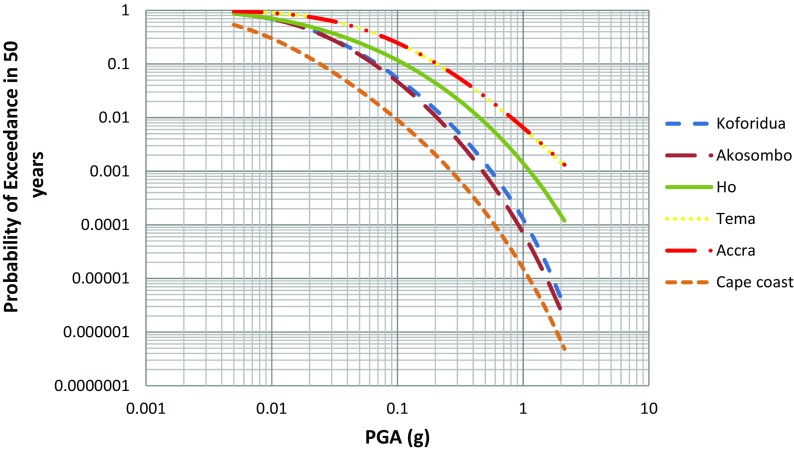
Seismic-hazard curves for the selected cities of specific site depicted in Table 4

**Fig. 11:**
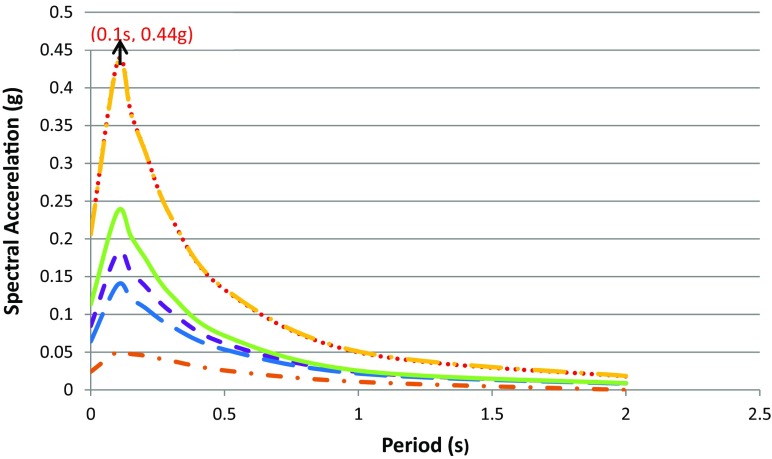
Uniform hazard spectra (return period of 475 year) for a proposed design spectrum for the cities considered

The hazard input parameters used in the calculation are listed in Table 3, and calculations were done for every 0.45°. The seismic hazard map obtained using the mean total hazard curve for every grid in the area of interest is shown in Fig. 9. An example of six hazard curves for specific sites and their corresponding uniform hazard spectra are shown in Figs. 10 and 11 respectively.

In the hazard map (Fig. 9), the highest levels of seismic hazard were found to occur in the Accra and Tema sub-zone, where peak ground accelerations (PGAs) in excess of 0.2 g are expected with 10% chance of exceedance in 50 years. The region with the next highest level of hazard is Ho sub-zones with PGA of 0.1 g. Koforidua and Cape Coast regions have PGA values of 0.08 and 0.026 g respectively.

The seismic hazard diminishes to a value of 0.05 g at a distance of 140 km from the highest hazard region (the threshold value of engineering interest) to the WNW direction. The hazard at six sites (Accra, Akosombo, Cape Coast, Ho, Koforidua and Tema) was also extracted and plotted as hazard curves (Fig. 10) and uniform hazard spectra obtained for 10% probability of exceedance in 50 years (Fig. 11). Shown in Table 4 are PGA values obtained at the six sites also for 10% probability of exceedance in 50 years (return period of 475 years).

**Table 4:** PGA values at rock level for six cities in southern Ghana

Cities	Location		PGA values (g)
	Longitude (°)	Latitude (°)	For return period 475 years
Accra	− 0.182	5.555	0.20
Akosombo	0.05	6.346	0.06
Cape Coast	− 1.255	5.1	0.026
Ho	0.408	6.649	0.10
Tema	0.035	5.657	0.20
Koforidua	− 0.226	6.068	0.08

**Fig. 12:**
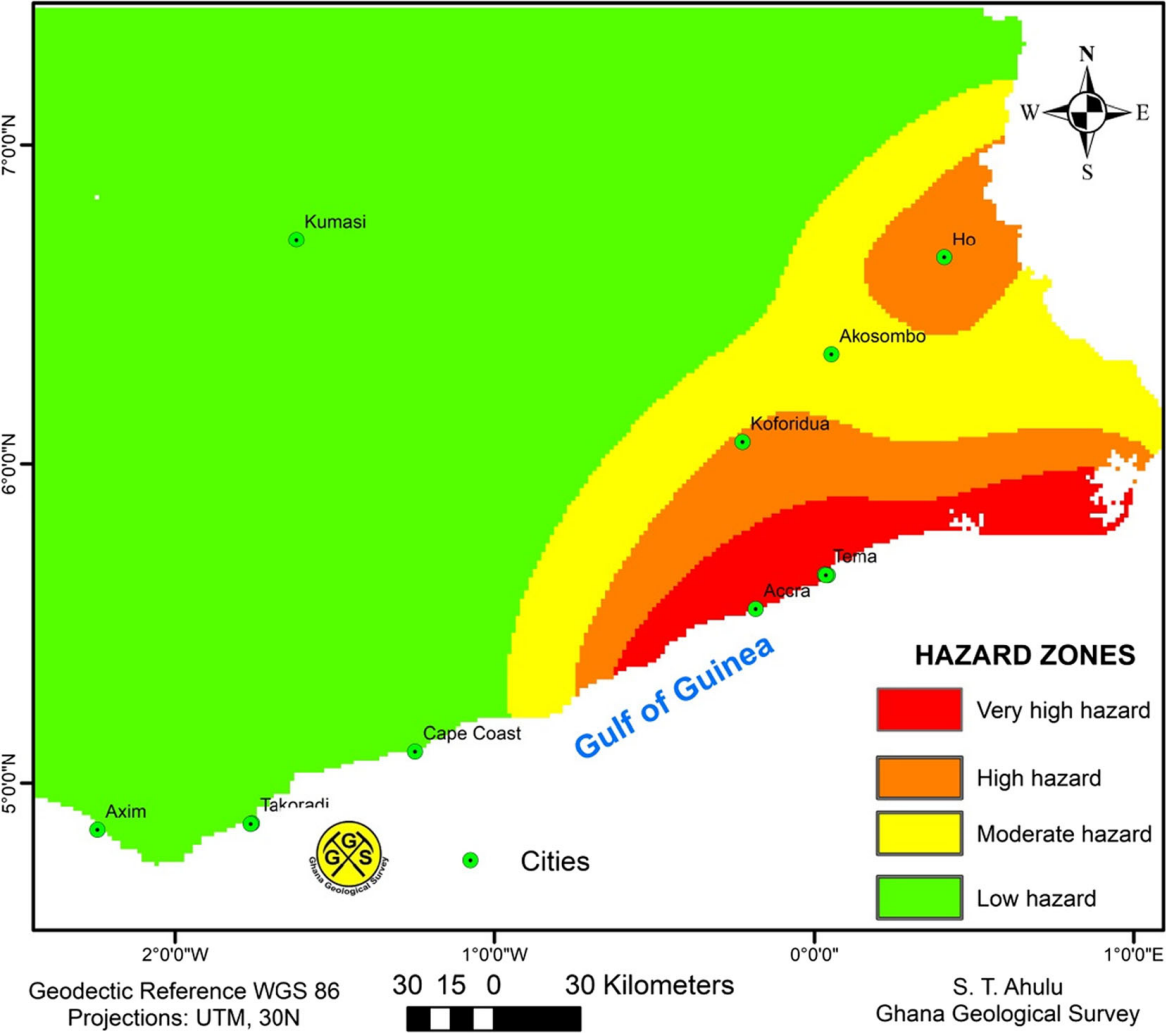
Seismic hazard zones for southern Ghana as obtained using the 10% probability of exceedance in the 50-year hazard map from this study

Four seismic hazard zones (Fig. 12) were identified in the southern part of Ghana based on the probabilistic seismic hazard analysis results. The seismic hazard zones are the following. Zone A (very high hazard): This largely covered Accra and Tema Metropolis where PGA values of approximately 0.2 g are expected with probability of 10% in 50 years. Zone B (high hazard): This includes the Metropolis of Koforidua and Ho. Zone C (moderate hazard): This includes the Akosombo District and surrounding area. Zone D (low hazard): This covers the rest of the southern part of Ghana.

Hazard curve is usually expected to be produced for different periods (i.e. for PGA, *T* = 0.1 s, 0.2 s, etc.). Each period has its own separate hazard curve. It also provides acceleration values for different return periods from each curve. However, hazard curves produced for this study are based on PGA.

From the hazard curve (Fig. 10), the PGA value of 0.2 g for Accra and Tema corresponds to probability of such events occurring to ~ 0.1, and is expected to be exceeded with probabilities of 10, 30 and 60% in 10, 50 and 100 years respectively. Thus, the probability of occurrence of such a likely “scenario earthquake” is moderate. In the same vein, if Accra and Tema zones are likely to experience 0.2 g every 10 years, then it means the acceleration to 475 years is high, and therefore Accra and Tema is a highly hazard zone.

The seismic hazard at sites (cities) was also presented in the form of a uniform hazard spectrum, which relates spectral period to acceleration (Fig. 11). It provides understanding in variation of acceleration with period and is very useful for engineers in the design of various structures as different structures sometime require acceleration at different periods. So in doing hazard assessment for a site, it is recommended to prepare uniform hazard spectra (UHS) that covers a range of periods required by engineers.

From the UHS curves (Fig. 11), cities with the highest computed seismic hazard value among the selected cities are Accra and Tema with mean PGA values of 0.2 g. The maximum spectral acceleration SA/max value observed at the two cities was 0.44 g for a return period of 475 years. In addition, it is observed that the SA/max value for all cities occurred at a natural period of about 0.1 s, which is the period meant for only one-storey buildings for the southern part of the Ghana territory.

## Discussions

A summary of peak ground acceleration values of some cities obtained in previous studies is shown in Table 5. Apart from Amponsah et al. (2009) who used the deterministic method and GSHAP (Grunthal et al. 1999) who used the probabilistic approach, almost all the previous studies done were based on the four revised seismic zones proposed by Anon. (1988) who used the 1939 isoseismal intensity scale pattern to designate the seismic zones, and assign PGA values. The probabilistic method used in this study incorporates the variability in different input parameters through a logic-tree approach in the computation of the seismic hazard.

**Table 5:** Comparison of hazard values obtained in the current study to those obtained in previous studies. *GSHAP* Global Seismic Hazard Assessment Program, *CSDCS* Code for Seismic Design of Concrete Structures, *BRRI* Building and Road Research Institute

Location	PGA values (g)				
	Present study	Previous studies			
Accra	0.20	0.14–0.57 Amponsah et al. (2009)	0.15 Kumapley (1996)	0.08–0.16 GSHAP (Grunthal et al. 1999)	0.35 CSDCS (BRRI 1990)
Weija (Accra—West)	0.20	0.2 Deakin (1941)	0.15 Kumapley (1996)	0.08 Anon. (1988)	0.35 CSDCS (BRRI 1990)
Ho	0.10		0.10 Kumapley (1996)	0.04 Anon. (1988)	0.25 CSDCS (BRRI 1990)
Cape Coast	0.026		0.15 Kumapley (1996)	0.02 Anon. (1988)	0.15 CSDCS (BRRI 1990)

The maximum PGA of 0.2 g for Accra obtained in the present study is comparable with the values of 0.16, 0.15 and 0.08 g suggested by Grunthal et al. (1999), Kumapley (1996) and Anon. (1988) respectively. For Accra city, the result from this study matches well with that from Deakin (1941) while that of Ho city matches well with that from Kumapley (1996). Under GSHAP, Grunthal et al. (1999) reported that the western part of Africa has a maximum PGA value of around 0.08 g with equatorial West Africa slightly higher at about 0.1 g. These values are comparable with our present study values. The PGA values of the rest of the cities whose previous studies were based on isoseismal intensity data do not match well with the present study values obtained.

There are large differences between the PGA values obtained using deterministic and probabilistic approaches. The deterministic approach is essentially based on a worst case scenario where the event occurs close to the site. In such a case, the deterministic approach is usually used for significant structures like power plants, large dams and large bridges. In developing such scenario earthquakes, considerations should be given to the potential fault locations which can generate these earthquakes. The two approaches can complement one another by providing different perspectives on the seismic hazard or risk problem.

The results obtained for Accra using the deterministic approach by Amponsah et al. (2009) gave a maximum PGA value of 0.57 g. However, the probabilistic hazard curve (Fig. 10) shows that the probability of occurrence of an event generating this much PGA in Accra is only 0.02. This equates to a very low probability event with a 2400-year return period.

A PGA value of 0.35 g was obtained for Accra using the isoseismal method (BRRI 1990). In this case, it is likely that amplification might have contributed to the PGA value. This is because most of the heavily damaged buildings were in Accra and were constructed on alluvium soil. And since the method used was based on damage classification, assigning slightly high PGA values for Accra would certainly have been influenced by the observed damage. Thus, comparing this value to that obtained in this present study, where hazard was calculated for a hard-rock site condition, is unreasonable.

## Conclusion

The present study has made an attempt to reach a significant improvement in seismic hazard assessment of the southern part of Ghana, with the aid of a new seismic source model. Moreover, well-known and reliable GMPEs were selected and incorporated into the hazard calculation using the logic-tree formalism.

The results are presented in the hazard map, and hazard curves for six cities as well as uniform hazard spectra for the same cities for 10% probability of exceedance in 50 years. According to this assessment, the highest seismic hazard values occur in the Greater Accra region which previously experienced the largest recorded Ghanaian earthquake (22 June 1936 Mw 6.4 event). Away from Accra, other significant seismic hazard areas are observed around Ho Metropolis, Koforidua Metropolis and Akosombo District. All are located at the north eastern side of Accra. These regions exhibit moderate to high earthquake activity, both in the historical and recent times, which in turn contributes to the seismic hazard.

Finally, when comparing the computed mean PGA values (for a return period of 475 years) of selected cities with previous studies, significant differences were noticed among values in the Code for Seismic Design of Concrete Structures (BRRI 1990), especially for some important cities (e.g. Accra).
